# The Use and Management of Invasive Plants

**DOI:** 10.3390/plants14071031

**Published:** 2025-03-26

**Authors:** Danijela Poljuha, Barbara Sladonja

**Affiliations:** Department of Agriculture and Nutrition, Institute of Agriculture and Tourism, Karla Huguesa 8, 52440 Poreč, Croatia; barbara@iptpo.hr

Invasive plant species pose significant challenges to biodiversity, ecosystem stability, and economic sustainability, such as disrupting native flora, altering soil chemistry, and creating ecological imbalances. However, recent research highlights the potential of these species as resources. Their secondary metabolites, allelopathic properties, and adaptability offer avenues for novel applications, particularly in phytopharmacy, energy production, and agriculture. This topical collection presents key advancements in both the control and utilization of invasive plant species, exploring their chemical properties, ecological impact, potential applications, and alternative management strategies.

Several studies in this collection investigate the bioactive compounds of invasive plants and their potential medicinal applications. One study focused on *Solidago canadensis* L., an invasive species in Europe, to investigate its total phenolics, flavonoids, non-flavonoids, and species-specific phenolic compounds. The findings indicated that phenolic acids and flavonoid-rich extracts from the invasive *S. canadensis* exhibit strong antioxidant properties, suggesting their potential in addressing oxidative-stress-related diseases [1]. In addition to the phytochemical properties of invasive alien plant species, their potential as antimicrobial agents is also being explored. Species such as *Ailanthus altissima*, *Ambrosia artemisiifolia*, *Conyza canadensis*, *Dittrichia viscosa*, *Erigeron annuus*, and *Xanthium strumarium* have shown significant antimicrobial activity. In one study, among all tested plant extracts, some effectively inhibited bacterial and fungal growth while exhibiting lower cytotoxicity compared to a positive control, with the exception of *X. strumarium* [2]. These findings suggest that invasive plants may be valuable sources of natural antimicrobials and pharmaceuticals. Further analysis of their phytochemicals also revealed potential cytotoxic effects against human cancer cells. *Acacia melanoxylon*, another invasive species, was studied to determine its chemical properties and cytotoxicity. The research identified five major compounds in *A. melanoxylon* extracts, including lupeol derivatives, which showed potential cytotoxic activity against colorectal cancer cells [3]. Another study showed that extracts of the highly invasive *Reynoutria japonica* rhizome exhibit promising activity against multidrug-resistant bacteria, such as *Mycobacterium smegmatis*. This plant is also an important source of stilbenes and anthranoids. The investigation concentrated on extraction methods, revealing that simple, food-grade solvent extractions can efficiently concentrate bioactive compounds, making them more viable for pharmacological use. The study highlighted *Reynoutria japonica*’s potential as an antimicrobial agent, suggesting alternative management strategies that incorporate its bioactive properties rather than focusing solely on eradication [4].

Understanding the biochemical strategies that enhance the invasiveness of plant species is crucial for effective management. Research in this area investigates how flavonoids and other secondary metabolites contribute to the competitive advantages of invasive plants. These compounds interact with soil nutrients and microbial communities, influencing root development and plant–microbe interactions. Some invasive species, such as *S. canadensis*, form nitrogen-containing complexes that regulate plant growth, even when released at very low concentrations, potentially altering local ecosystems and promoting further invasion [5]. Managing invasive species often involves costly eradication efforts with limited success. This collection presents alternative management strategies, including biomass utilization and microbial-based control. The highly toxic species *Jacobaea vulgaris* has been studied to determine the energetic potential of its biomass through biomethanization, demonstrating that ensiling can significantly reduce its toxic alkaloid content, making this invasive species safer for potential use in bioenergy production. Biomethanization experiments revealed that the biomasses of some invasive plants, though limited in terms of energy potential, could still provide sustainable alternatives for waste management and renewable energy [6].

The review articles in this collection provide a broader context for managing invasive plants, integrating biological control, chemical utilization, and novel eradication techniques. *Senecio madagascariensis* is an exotic invasive species that poses a major threat to natural and modified ecosystems around the world. The review presented in [7] synthesizes the currently available information on the biology, distribution, and management options of *S. madagascariensis*’s, as well as on its ecological impact. It also discusses integrated management strategies, including biological control and competitive planting, as part of a systematic approach. Another review on *Fallopia japonica* provides an overview of its high therapeutic potential, highlighting its antioxidant, antimicrobial, anti-inflammatory, and anticancer effects. Among the key biological compounds found in this species are resveratrol, emodin, and polydatin. The review also explores its potential for honey production, offering an innovative perspective on its economic value [8]. Other studies explore microbial-based control methods, emphasizing the role of plant–microbe interactions in suppressing invasive growth while supporting native plant species. One review highlights the increased research into microbial-mediated phytochemical production in invasive plants, microbial consortia that reduce the competitiveness of invasive plants, microbial-mediated increases in the herbicidal tolerance of native plants, and the increased susceptibility of invasive plants to plant pathogens [9]. Despite numerous novel control and eradication methods, classic mechanical methods can still be valuable, especially if key factors for their successful implementation are identified. Managing invasive exotic plant species is a complex challenge, particularly for Asian knotweeds (*Reynoutria* spp.). Tarping is a common but poorly documented method of managing this species, and the review presented in [10] emphasizes the importance of site-specific conditions and long-term monitoring for its successful implementation. Moreover, based on the review’s bibliography and survey work, practical recommendations are proposed.

This topical collection demonstrates the complex nature of invasive plant research, emphasizing the importance of viewing these species not only as ecological threats but also as potential resources. The work reported in this collection of papers will contribute to a growing body of evidence that invasive plants can be managed in ways that promote sustainability, whether through pharmaceutical applications, bioenergy production, or alternative control strategies. Future research should continue to explore these dual perspectives, ensuring that ecological balance is maintained while maximizing the potential benefits of invasive species.
